# Supplementary material for: Environmental fungi target thiol homeostasis to compete with Mycobacterium tuberculosis
Source: PLoS Biol. 2024 Dec 3;22(12):e3002852. doi: 10.1371/journal.pbio.3002852 (PMC11614215; doi:10.1371/journal.pbio.3002852)

**S1 Test:** Supplemental chemistry section

^1^H and ^13^C NMR spectra were recorded on a Varian Mercury-400 NMR Spectrometer and 2D NMR spectra on a Varian Mercury-600 NMR Spectrometer. The chemical shifts were measured in ppm relative to chloroform solvent signal (δ 7.26) or DMSO (δ 2.50). Routine mass analyses (LRMS) were performed on HP Agilent LC/MS series 1100 system equipped with a reverse phase column (Agilent Poroshell 120 EC-C18, 2.7 μm, 50 × 2.1 mm) and photodiode array detector using electrospray ionization (ESI). Accurate masses (HRMS) were obtained using Waters LCT Premiere time-of-flight mass spectrometer. The instrument was operated in the W-mode at a resolution of 10,000 and in positive ion mode. Ions were generated with the Z-Spray electrospray ionization (ESI) with a capillary voltage of 3.4 kV. Accurate masses were determined using the internal standard method. The metabolite was monitored using thin-layer chromatography (TLC) (silica gel 60 F254 0.25 mm), and the products were visualized by UV light (254 nm). SiliaFlash® P60 (40–60 µm) used in flash column chromatography was purchased from Silicycle Inc.

3-1. (−)-Nidulain A ^3,4^

R*f* = 0.44 (EA/Hexane = 1/1); [α]D = −578.5 (c = 1.0, CHCl_3_, at 25°C); ^1^H NMR (400 MHz, CDCl_3_) δ 12.14 (s, 1H, **8**-OH), 7.36 (dd, *J* = 2.3 and 4.3 Hz, 1H, **1**-H), 6.44−6.40 (m, 3H, **2**-, **3**-, **7**-H), 6.34 (s, 1H, **5**-H), 4.69 (d, *J* = 4.6 Hz, 1H, **4**-H), 3.65 (s, 3H, **4a**-CO_2_Me), 2.60 (bs, 1H, **4**-OH), 2.29 (s, 3H, **6**-Me); ^13^C NMR (100 MHz, CDCl_3_) δ 182.9 (**9**-C), 169.0 (**4a**-*C*O_2_Me), 163.0 (**8**-C), 158.1 (**10a**-C), 151.1 (**6**-C), 131.85 (**3**-C), 131.75 (**1**-C), 127.3 (**9a**-C), 126.6 (**2**-C), 111.6 (**5**-C), 108.5 (**7**-C), 105.8 (**8a**-C), 83.1 (**4a**-C), 65.3 (**4**-C), 53.6 (**4a**-CO_2_*Me*), 22.7 (**6**-Me); LRMS (ESI) m/z 303 [M + 1]^+^.

3-1-1. ^1^H NMR of (−)-Nidulain A


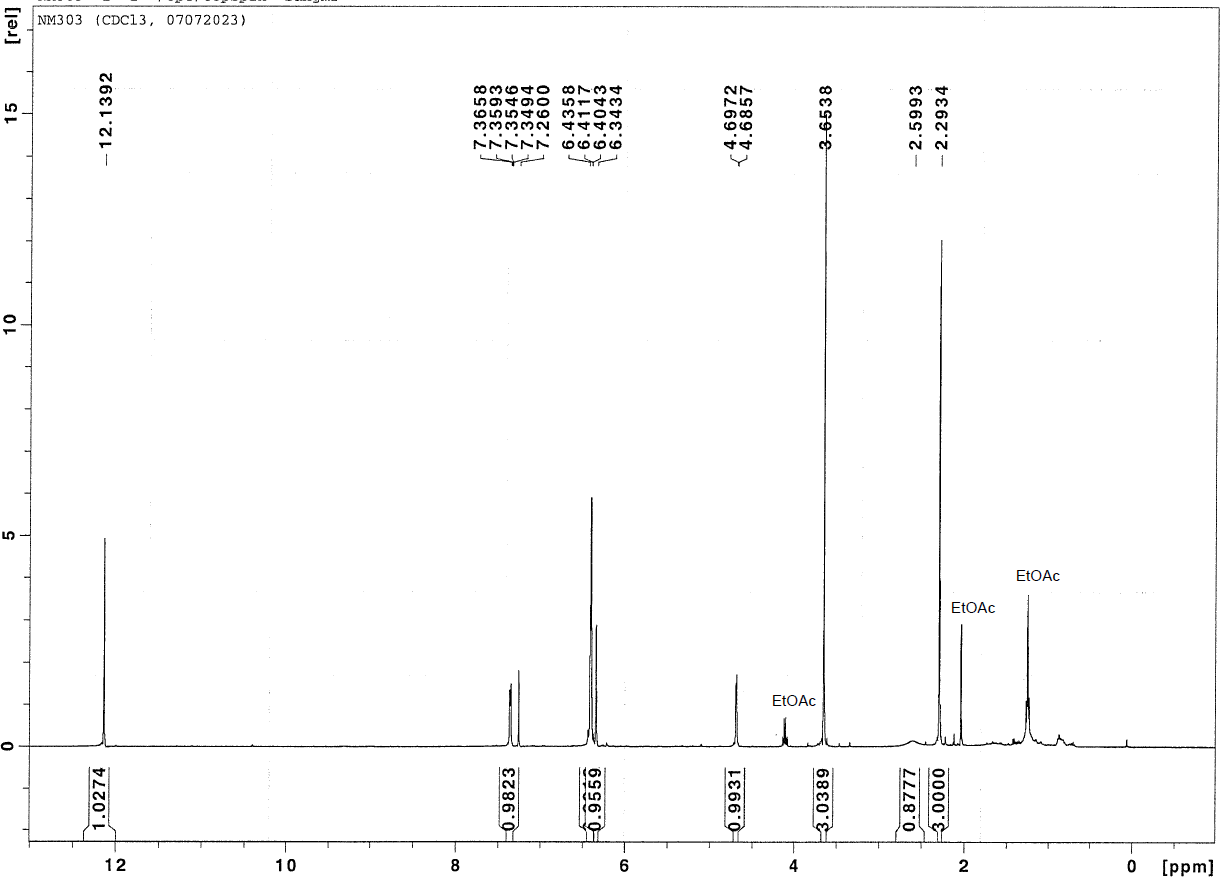


3-1-2. ^13^C NMR of (−)-Nidulain A


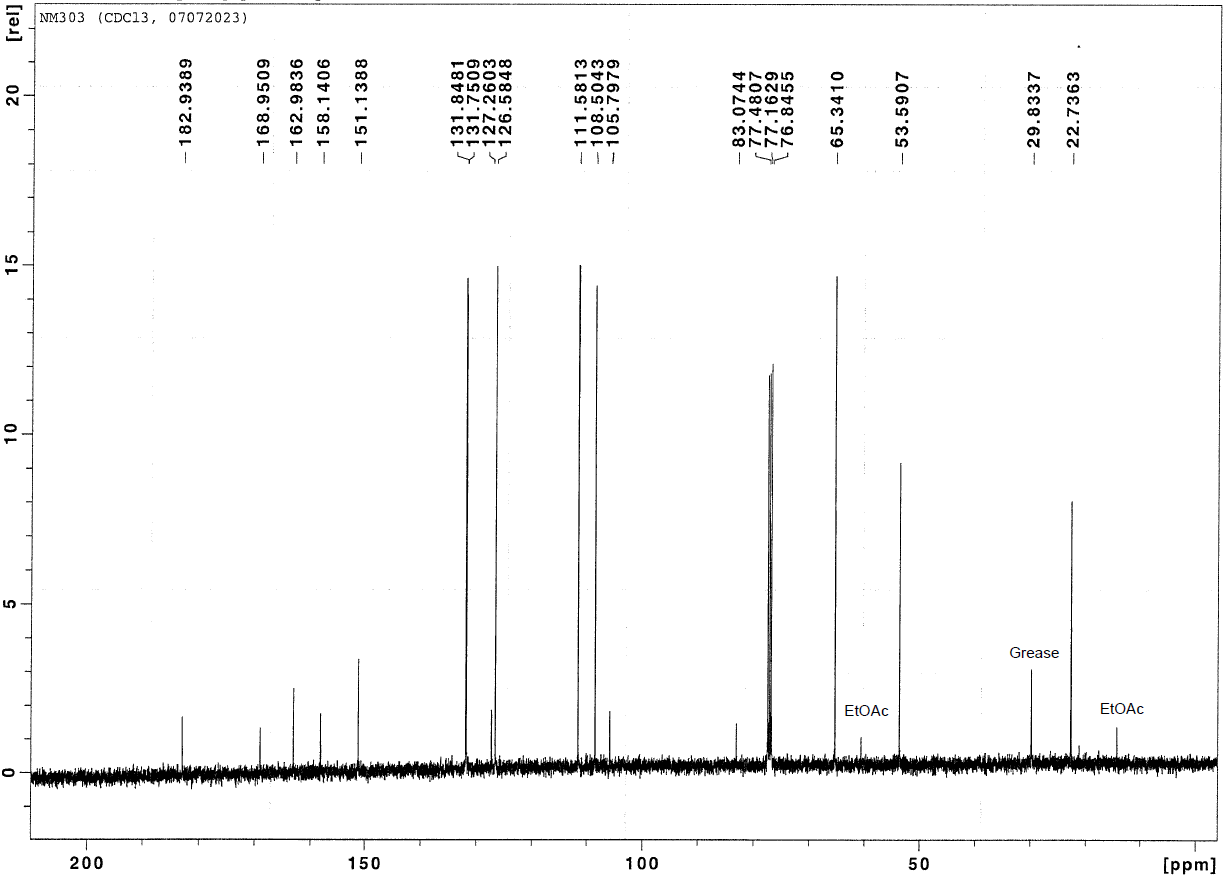


3-2. Citrinin ^5^

R*f* = 0.23 (only EA); ^1^H NMR (400 MHz, DMSO-*d^6^*) δ 16.3 (bs, 1H, **7**-COOH), 15.40 (bs, 1H, **8**-OH), 8.62 (s, 1H, **1**-H), 4.99 (q, *J* = 6.6 Hz, 1H, **3**-H), 3.21 (q, *J* = 7.2 Hz, 1H, **4**-H), 1.96 (s, 3H, **5**-Me), 1.26 (d, *J* = 6.6 Hz, 3H, **3**-Me), 1.12 (d, *J* = 7.2 Hz, 3H, **4**-Me); ^13^C NMR (100 MHz, DMSO-*d^6^*) δ 183.2 (**6**-C), 176.4 (**8**-C), 174.1 (**7**-*C*OOH), 166.8 (**1**-C), 141.1 (**4a**-C), 121.3 (**8a**-C), 106.5 (**5**-C), 99.4 (**7**-C), 82.0 (**3**-C), 33.5 (**4**-C), 18.0 (**5**-Me), 17.5 (**3**-Me), 9.0 (**4**-Me); HRMS (ESI) m/z calculated for C_13_H_15_O_5_ [M + H]^+^ 251.0919, found 251.0919.

3-2-1. ^1^H NMR of Citrinin


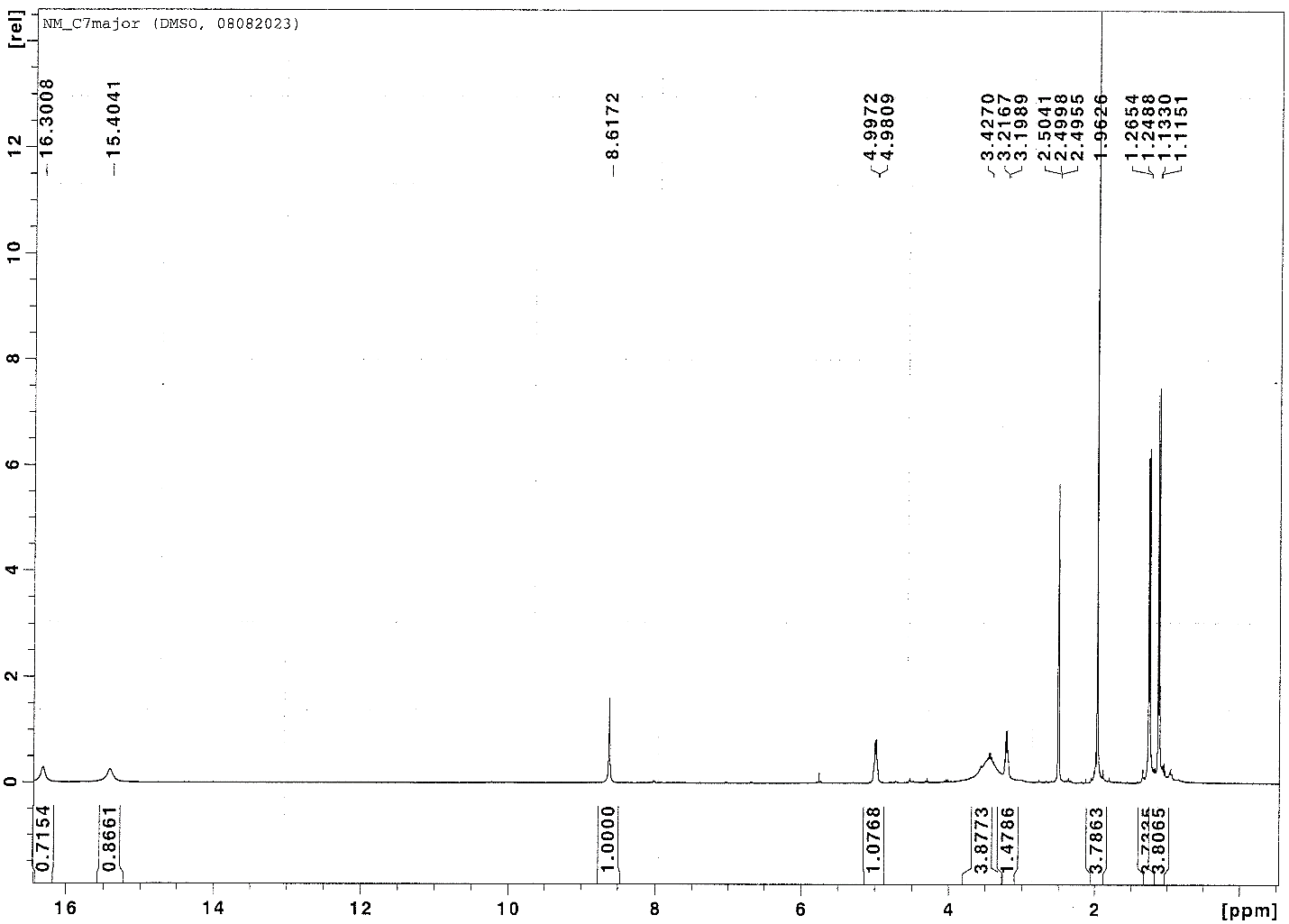


3-2-2. ^13^C NMR of Citrinin


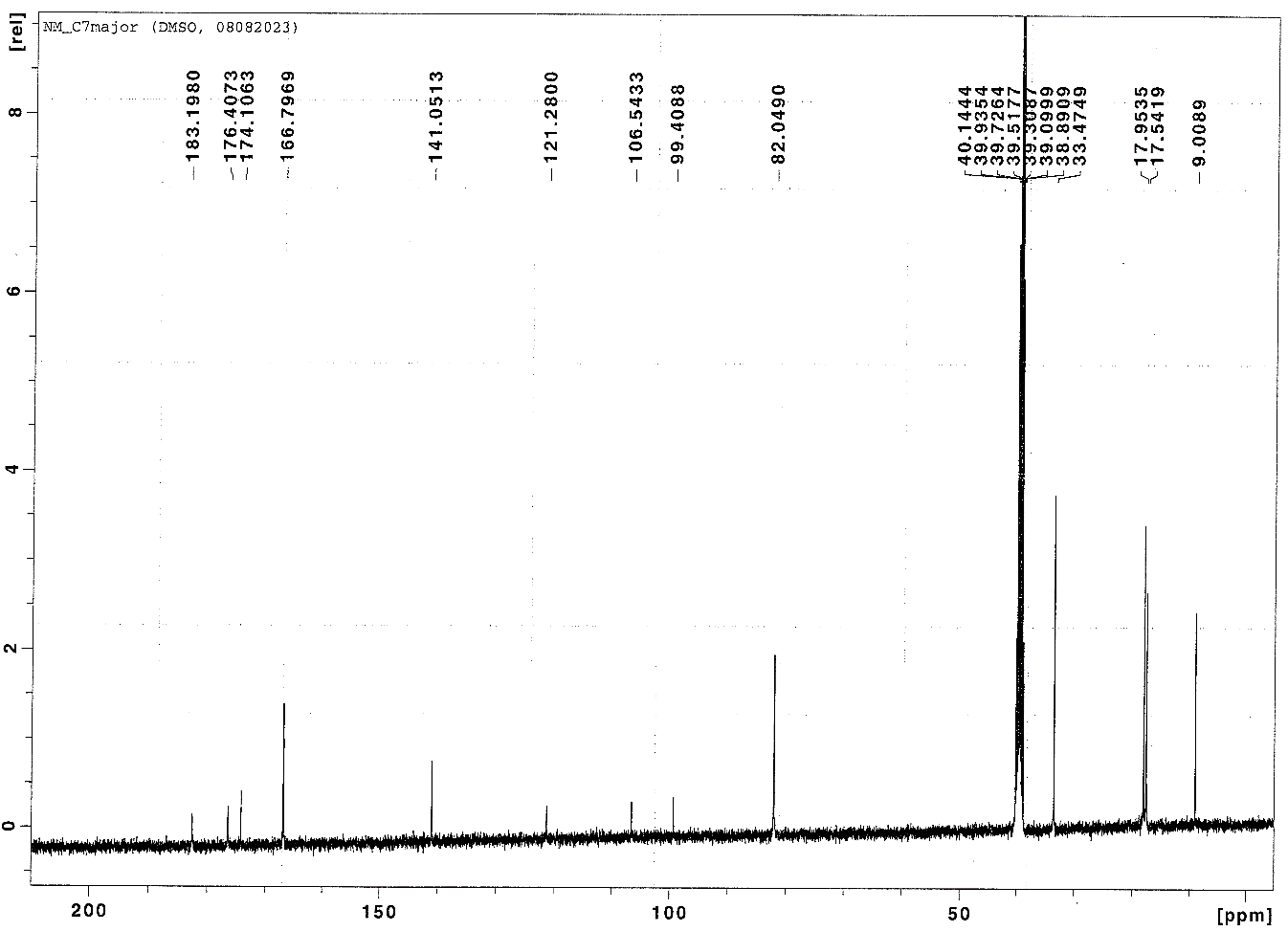


3-2-3. HSQC of Citrinin


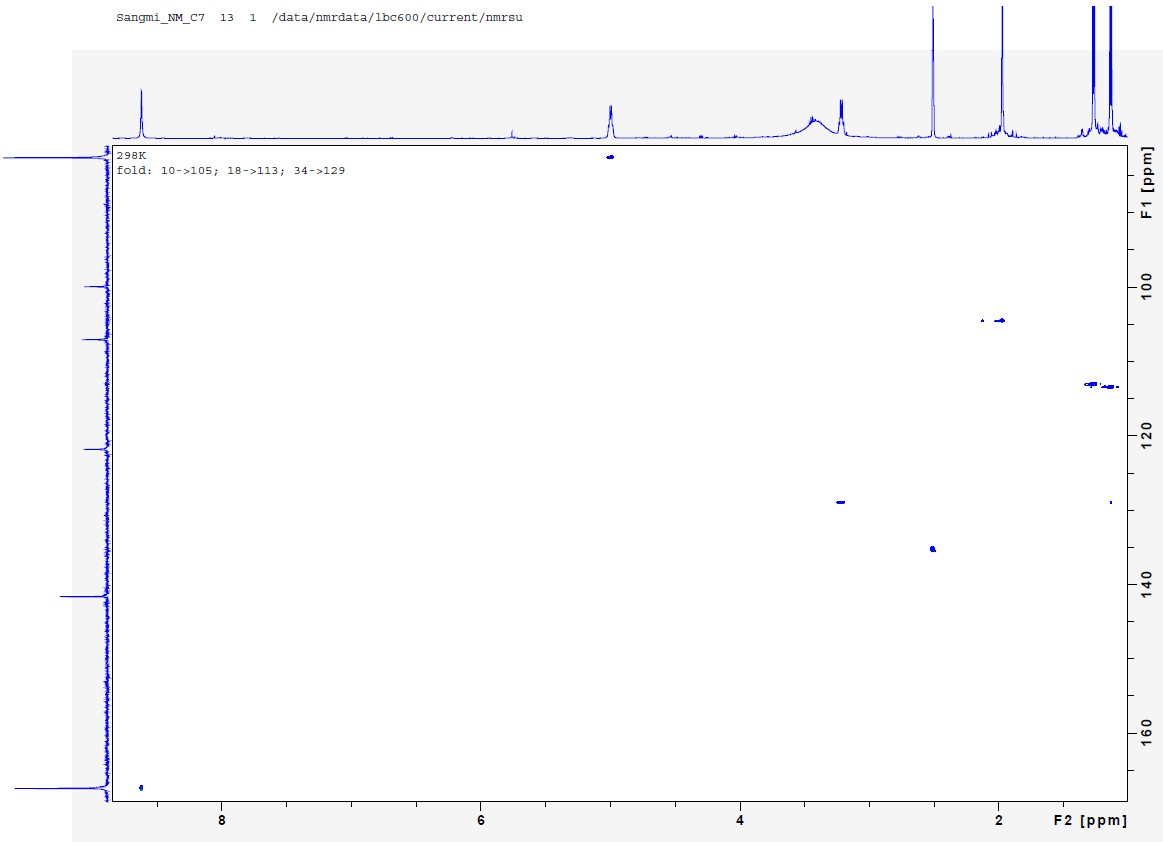


3-2-4. HMBC of Citrinin


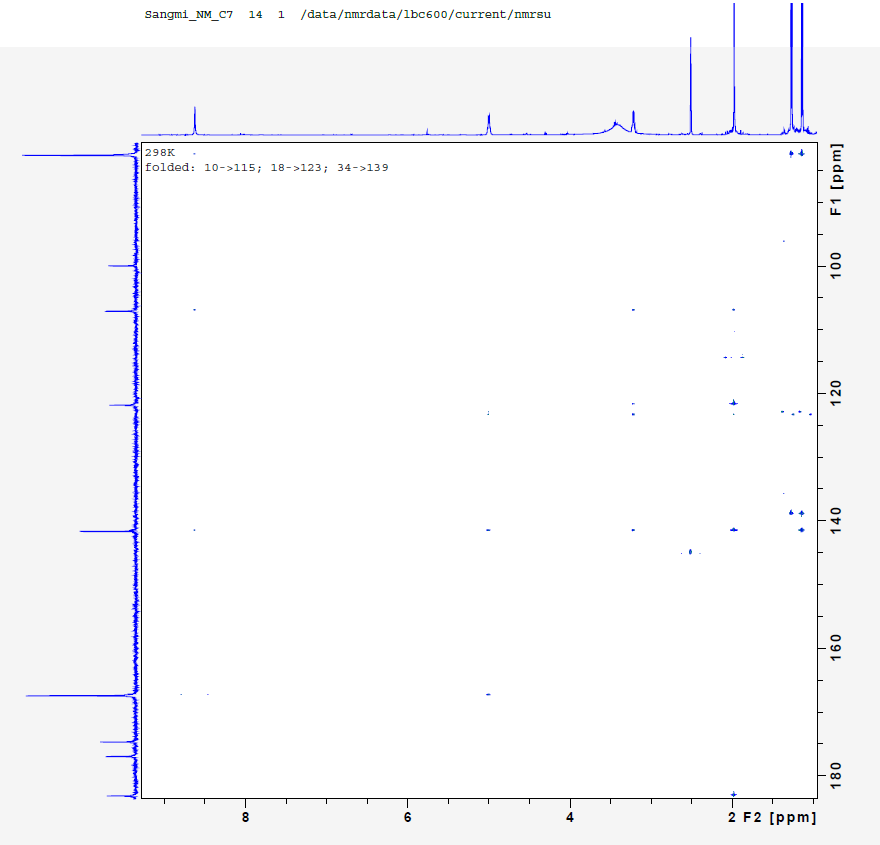

Supplement: S1 Text — (DOCX) [file pbio.3002852.s004.docx]
