# Supplementary material for: Environmental fungi target thiol homeostasis to compete with Mycobacterium tuberculosis
Source: PLoS Biol. 2024 Dec 3;22(12):e3002852. doi: 10.1371/journal.pbio.3002852 (PMC11614215; doi:10.1371/journal.pbio.3002852)
Supplement: S1 Table — (DOCX) [file pbio.3002852.s005.docx]

**S1 Table:** Primers used in the study

| **Primer name** | **Sequence** | **PCR target** |
| --- | --- | --- |
|  |  |  |
| ITS5F | 5’ GGAAGTAAAAGTCGTAACAAGG | ITS1 region |
| ITS4R | 5’ TCCTCCGCTTATTGATATGC | ITS1 region |
| 16s-27 5’F | 5’ GTTTGATCVTGGCTCAG | 16s region |
| 16s-80 5’R | 5’ CTACHVGGGTATCTAATCC | 16s region |
| C7_120a 5’F | ATGCCTGACTCAACTTCTCACCCACACC | C7_120 Type I PKS |
| C7_120a 5’R | TCTGTATCCTCCGTAATGGACTTGGAC | C7_120 Type I PKS |
| C7_116 5’F | ATGGCCGAAACAGCAACTGAAATTC | *dehydrogenase* |
| C7_116 5’R | TTGCCCGCTGCTAGACATGGC | *dehydrogenase* |
| C7_118 5’F | ATGCCCGTACCAACCAAGTCATC | *dioxygenase* |
| C7_118 5’R | TCACTTTGCCTTGGTGTTATAAGTG | *dioxygenase* |
| oPMF369 | CTCCCACCTGCGTGC**CGGT**TTGCGTGCCGCGTCGC | *Rv3054c* upstream region |
| oPMF370 | CGGACACCTGCTCTG**TCAT**TGCTACGTTCCTTTCCGCTT | *Rv3054c* upstream region |
| oPMF371 | TAAGCACCTGCCTCC**ATGA**GCGGTCACCATCACC | mScarlet |
| oPMF372 | TTTTCACCTGCAGCT**CAAG**CTTGTACAGCTCGTCCATGCC | mScarlet |
| oPMF373 | AGCACACCTGCGAGG**CTTG**GCTCGAAGTGCAGGA | Entire plasmid |
| oPMF374 | TGGTCACCTGCACTC**ACCG**GGTTGTTGACACCG | Entire plasmid |

* 5’ overhangs are in red; *PaqCI* recognition sites are underlined, and the 4 bp overhangs generated by *PaqCI* digestion are indicated in bold.
