# Supplementary material for: Environmental fungi target thiol homeostasis to compete with Mycobacterium tuberculosis
Source: PLoS Biol. 2024 Dec 3;22(12):e3002852. doi: 10.1371/journal.pbio.3002852 (PMC11614215; doi:10.1371/journal.pbio.3002852)
Supplement: S2 Table — (DOCX) [file pbio.3002852.s006.docx]

**S2 Table:** MIVs (µL) for F2+*Mtb*, C7+*Mtb* and F31+*Mtb* filtrates against different bacteria

|  | **F2 + *Mtb* (μL)** | **C7 + *Mtb* (μL)** | **F31 + *Mtb* (μL)** |
| --- | --- | --- | --- |
| *Mtb* | 1.5 | 12.5-25.0 | 1.5-3.15 |
| *M.bovis* BCG | 3.15 | 12.5-25.0 | 3.15 |
| *M.smegmatis* | >50 | 50 | >50 |
| *Enterococcus faecalis* | >50 | >50 | >50 |
| *Staphylococcus aureus* | >50 | 50 | >50 |
| *Klebsiella pneumoniae* | >50 | >50 | >50 |
| *Acinetobacter baumannii* | >50 | >50 | >50 |
| *Pseudomonas aeruginosa* | >50 | >50 | >50 |
| *Enterobacter sp.* | >50 | >50 | >50 |

*Fungal filtrates used for this assay were obtained from co-cultures on PDB media and have not been concentrated by lyophilization. Same set of filtrates were used for testing MIV against all bacteria tested.
