# Supplementary material for: Environmental fungi target thiol homeostasis to compete with Mycobacterium tuberculosis
Source: PLoS Biol. 2024 Dec 3;22(12):e3002852. doi: 10.1371/journal.pbio.3002852 (PMC11614215; doi:10.1371/journal.pbio.3002852)
Supplement: S3 Table — (DOCX) [file pbio.3002852.s007.docx]

**S3 Table:** Differential expression of F31 gene cluster in co-culture conditions and their amino acid identity with proteins belonging to agnestin or neosartorin pathway

| **Gene ID** | **log_2_FC** | **Padj** | **F31 - *Mtb*** | **F31 + *Mtb*** | **Best Match** | **Identity** | **Function** |
| --- | --- | --- | --- | --- | --- | --- | --- |
| **F31_005358** | -3.81 | 1E-38 | 547 | 4398 | NsrC | 74% | Thioesterase (Metallo-betalactamase) |
| **F31_005359** | -3.88 | 1.6E-45 | 350 | 2819 | NsrE | 80% | Decarboxylase |
| **F31_005360** | -3.96 | 9.1E-50 | 494 | 4259 | NsrF | 60% | Baeyer-villager oxidase |
| **F31_005361** | -3.06 | 3.6E-13 | 90 | 326 | NsrQ | 39% | Monooxygenase |
| **F31_005362** | -3.64 | 2.2E-30 | 174 | 1077 | AgnL4 | 66% | Oxidoreductase |
| **F31_005363** | -3.54 | 1.0E-38 | 1905 | 13733 | AgnL5 | 68% | Short-chain dehydrogenase |
| **F31_005364** | -3.45 | 4.7E-35 | 930 | 6272 | AgnL8 | 61% | Dehydratase |
| **F31_005365** | -3.20 | 2.3E-25 | 195 | 971 | NsrG | 57% | Methyltransferase |
| **F31_005366** | -2.70 | 2.1E-27 | 2115 | 9453 | TF | 69% | Zn(2)-C6 TF |
| **F31_005367** | -3.15 | 6.8E-25 | 11944 | 71351 | MFS | 99% | MFS transporter |
| **F31_005368** | -4.20 | 2.5E-41 | 983 | 10196 | ? | 89% | Short-chain dehydrogenase |
| **F31_005369** | -4.08 | 1.9E-61 | 440 | 4017 | NsrK | 60% | FAD-dependent monooxygenase |
| **F31_005370** | -3.31 | 9.3E-25 | 118 | 547 | TF | 91% | Regulatory protein |
| **F31_005371** | -1.18 | 0.06 | 503 | 932 | - | - | Hypothetical |
| **F31_005372** | -4.16 | 1.8E-42 | 1806 | 19015 | NsrB | 58% | Non-Reducing PKS |

*Nsr–Neosartorin biosynthesis enzymes^1^ ; **AgnL–Agnestin biosynthesis enzyme^2^; ‘-’ refers to very small size protein with no close homologs in any pathways; ‘?’ refers to no close functional homolog in either agnestin or neosartorin pathway.
