# Supplementary material for: Environmental fungi target thiol homeostasis to compete with Mycobacterium tuberculosis
Source: PLoS Biol. 2024 Dec 3;22(12):e3002852. doi: 10.1371/journal.pbio.3002852 (PMC11614215; doi:10.1371/journal.pbio.3002852)
Supplement: S4 Table — (DOCX) [file pbio.3002852.s008.docx]

**S4 Table:** Differential expression of *Mtb* genes in response to F2+*Mtb* and F31+*Mtb* filtrates

| **Criteria** | **6h,1X** | **6h,10X** | **12h,1X** | **12h,10X** |
| --- | --- | --- | --- | --- |
|  |  |  |  |  |
| **F31 + *Mtb* log_2_FC>=1.0** | 87 | 836 | 54 | 850 |
| **F31 + *Mtb* log_2_FC <=1.0** | 78 | 805 | 57 | 968 |
| **F31 + *Mtb* log_2_FC >=2.0** | 13 | 353 | 8 | 378 |
| **F31 + *Mtb* log_2_FC <=2.0** | 5 | 197 | 5 | 334 |
| **F31 + *Mtb* log_2_FC >=3.0** | 4 | 194 | 4 | 191 |
| **F31 + *Mtb* log_2_FC <=3.0** | 0 | 43 | 0 | 98 |
| **F31 + *Mtb* log_2_FC >=4.0** | 2 | 103 | 1 | 95 |
| **F31 + *Mtb* log_2_FC <=4.0** | 0 | 15 | 0 | 32 |
|  |  |  |  |  |
| **F2 + *Mtb* log_2_FC >=1.0** | 95 | 839 | 70 | 948 |
| **F2 + *Mtb* log_2_FC <=-1.0** | 65 | 795 | 67 | 963 |
| **F2 + *Mtb* log_2_FC >=2.0** | 20 | 351 | 9 | 457 |
| **F2 + *Mtb* log_2_FC <=-2.0** | 6 | 164 | 5 | 351 |
| **F2 + *Mtb* log_2_FC >=3.0** | 3 | 185 | 5 | 236 |
| **F2 + *Mtb* log_2_FC <=-3.0** | 0 | 46 | 0 | 105 |
| **F2 + *Mtb* log_2_FC >=4.0** | 1 | 103 | 1 | 127 |
| **F2 + *Mtb* log_2_FC <=-4.0** | 0 | 16 | 0 | 40 |
|  |  |  |  |  |
