# Supplementary material for: Environmental fungi target thiol homeostasis to compete with Mycobacterium tuberculosis
Source: PLoS Biol. 2024 Dec 3;22(12):e3002852. doi: 10.1371/journal.pbio.3002852 (PMC11614215; doi:10.1371/journal.pbio.3002852)
Supplement: S5 Table — (DOCX) [file pbio.3002852.s009.docx]

**S5 Table:** MICs (µM) for nidulalin A, patulin and citrinin against *Mtb* H37Rv in different media

| **Compound** | **1-week MIC 7H9/GCAS/Tx** | **2-week MIC 7H9/GCAS/Tx** | **1-week MIC 7H9/ADC/Tw** | **2-week MIC 7H9/ADC/Tw** |
| --- | --- | --- | --- | --- |
| **Nidulalin A** | 1.56 | 2.3 | 1.4 | 1.4 |
| **Patulin** | 4.7 | 4.7 | 12.5 | 12.5 |
| **Citrinin** | >50 | >50 | >50 | >50 |
| **INH** | 0.049 | 0.07 | 0.1 | 0.15 |

*GCAS: Glucose, Casitone; Tx: Tyloxapol; Tw: Tween 80; INH: Isoniazid
