# Supplementary material for: Environmental fungi target thiol homeostasis to compete with Mycobacterium tuberculosis
Source: PLoS Biol. 2024 Dec 3;22(12):e3002852. doi: 10.1371/journal.pbio.3002852 (PMC11614215; doi:10.1371/journal.pbio.3002852)
Supplement: S6 Table — (DOCX) [file pbio.3002852.s010.docx]

**S6 Table:** Differential expression of patulin cluster in F2, F50 and F51 upon co-cultivation and their sequence identity with their homologs in *P.expansum*.

| **F2 Fungus Gene ID** | **log_2_FC** | **Padj** | **F2-*Mtb*** | **F2+*Mtb*** | **Identity** | **Functional Homologs** |
| --- | --- | --- | --- | --- | --- | --- |
| F2**_**003997 | -3.53 | 1.9E-10 | 91 | 444 | 91% | PKS-Enoyl Reductase (patD) |
| F2**_**003998 | -4.57 | 4.8E-31 | 1336 | 14215 | 83% | Drug Transporter (patC) |
| F2**_**003999 | -3.39 | 8.1E-22 | 172 | 896 | 79% | Carboxylesterase (patB) |
| F2_004001 | -2.32 | 2.7E-04 | 176 | 525 | 83% | ABC transporter (patM) |
|  |  |  |  |  |  |  |
| **F50 Fungus Gene ID** | **log_2_FC** | **Padj** | **F50-*Mtb*** | **F50+*Mtb*** | **Identity** | **Functional Homologs** |
| F50**_**006627 | -6.61 | 9.0E-166 | 313 | 14772 | 89% | Cytochrome P450 (patI) |
| F50**_**006628 | -6.20 | 8.7E-162 | 245 | 8811 | 89% | Hypothetical/Cupin-like (patJ) |
| F50**_**006629 | -9.02 | 0 | 788 | 156045 | 84% | Type I Polyketide Synthase (patK) |
| F50**_**006630 | -7.56 | 5.3E-174 | 239 | 19248 | 88% | Cytochrome P450 (patH) |
| F50**_**006631 | -7.40 | 2.0E-180 | 150 | 10054 | 88% | Hypothetical (patG) |
| F50**_**006632 | -8.05 | 1.1E-162 | 73 | 5079 | 81% | Hypothetical (patF) |
| F50**_**006633 | -5.15 | 4.3E-103 | 458 | 9491 | 88% | Hypothetical (patE) |
| F50**_**006634 | -5.60 | 1.8E-86 | 1425 | 41089 | 91% | PKS-Enoyl Reductase (patD) |
| F50**_**006635 | -4.03 | 1.6E-79 | 11383 | 126313 | 83% | Drug Transporter (patC) |
| F50**_**006636 | -4.26 | 5.6E-58 | 497 | 6135 | 79% | Carboxylesterase (patB) |
| F50**_**006637 | -6.87 | 6.3E-21 | 1780 | 113402 | 83% | ABC transporter (patM) |
| F50**_**006638 | -7.01 | 9.9E-224 | 345 | 20603 | 93% | SDR (patN) |
| F50**_**006639 | -6.63 | 3.4E-145 | 103 | 3967 | 74% | FAD-linked oxidase (patO) |
|  |  |  |  |  |  |  |
| **F51 Fungus**  **Gene ID** | **log_2_FC** | **Padj** | **F51-*Mtb*** | **F51+*Mtb*** | **Identity** | **Functional Homologs** |
| F51_008682 | -2.90 | 6.7E-38 | 335 | 1710 | 88% | Cytochrome P450 (patI) |
| F51_008683 | -2.04 | 7.3E-10 | 208 | 604 | 89% | Hypothetical/Cupin-like (patJ) |
| F51_008684 | -3.28 | 4.0E-51 | 1925 | 13580 | 84% | Type I Polyketide Synthase (patK) |
| F51_008685 | -3.73 | 5.7E-59 | 1779 | 16502 | 89% | Cytochrome p450 (patH) |
| F51_008686 | -4.28 | 9.3E-80 | 304 | 3513 | 88% | Hypothetical (patG) |
| F51_008687 | -2.72 | 4.6E-19 | 242 | 1083 | 81% | Hypothetical (patF) |
| F51_008688 | -2.70 | 7.6E-38 | 1066 | 5202 | 88% | Hypothetical (patE) |
| F51_008689 | -3.29 | 5.8E-70 | 5801 | 41570 | 91% | PKS-Enoyl Reductase (patD) |
| F51_008690 | -4.25 | 3.9E-131 | 13521 | 172007 | 83% | Drug Transporter (patC) |
| F51_008691 | -3.31 | 1.9E-62 | 489 | 3284 | 79% | Carboxylesterase (patB) |
| F51_008692 | -3.82 | 1.8E-08 | 231 | 2045 | 98% | Acetate transporter (patA) |
| F51_008693 | -4.81 | 4.6E-149 | 5407 | 95917 | 83% | ABC transporter (patM) |
| F51_008694 | -3.29 | 3.9E-46 | 1858 | 13260 | 93% | SDR (patN) |
|  |  |  |  |  |  |  |
