# Supplementary material for: Environmental fungi target thiol homeostasis to compete with Mycobacterium tuberculosis
Source: PLoS Biol. 2024 Dec 3;22(12):e3002852. doi: 10.1371/journal.pbio.3002852 (PMC11614215; doi:10.1371/journal.pbio.3002852)
Supplement: S7 Table — (DOCX) [file pbio.3002852.s011.docx]

**S7 Table:** Source Information for fungal hits identified in the study

| **Fungus ID** | **Source** | **Coordinates (lat, long)** | **Original media for isolation** | **Collection Date** | **Depth (Inches)** |
| --- | --- | --- | --- | --- | --- |
| F2 | Monogahela National Forest,  Cranberry Glades Botanical Area, WV, USA | 38.19993, -80.2640408 | PDA | 2017 | 22-24 |
| C7 | Quincy Bog, Rumney, NH, USA | 43.79271, -71.77730 | PDA | 2006 | Top layer |
| F31 | Monogahela National Forest,  Cranberry Glades Botanical Area, WV, USA | 38.19993, -80.26404 | Pectin | 2017 | 7-9 |
| F50 | Hobart Bog  Maine, USA | 44.850652, -67.254430 | PDA | 2018 | 10-13 |
| F51 | Hobart Bog  Maine, USA | 44.850652, -67.254430 | PDA | 2018 | 10-13 |
| G7 | Monogahela National Forest,  Cranberry Glades Botanical Area, WV, USA | 38.19993, -80.2640408 | PDA | 2017 | 39-41 |
| G9 | Monogahela National Forest,  Cranberry Glades Botanical Area, WV, USA | 38.19993, -80.2640408 | PDA | 2017 | 1-4 |
| D6 | Monogahela National Forest,  Cranberry Glades Botanical Area, WV, USA | 38.19993, -80.2640408 | PDA | 2017 | 1-3 |
| D9 | Sunkhaze bog  Maine, USA | 45.010357, -68.548442 | Sphagnum | 2018 | Top Layer |

*pH of PDA media: 5.4; Pectin media: 4.0; Sphagnum media: 4.0
