# Supplementary figures and images for: Environmental fungi target thiol homeostasis to compete with Mycobacterium tuberculosis
Source: PLoS Biol. 2024 Dec 3;22(12):e3002852. doi: 10.1371/journal.pbio.3002852 (PMC11614215; doi:10.1371/journal.pbio.3002852)

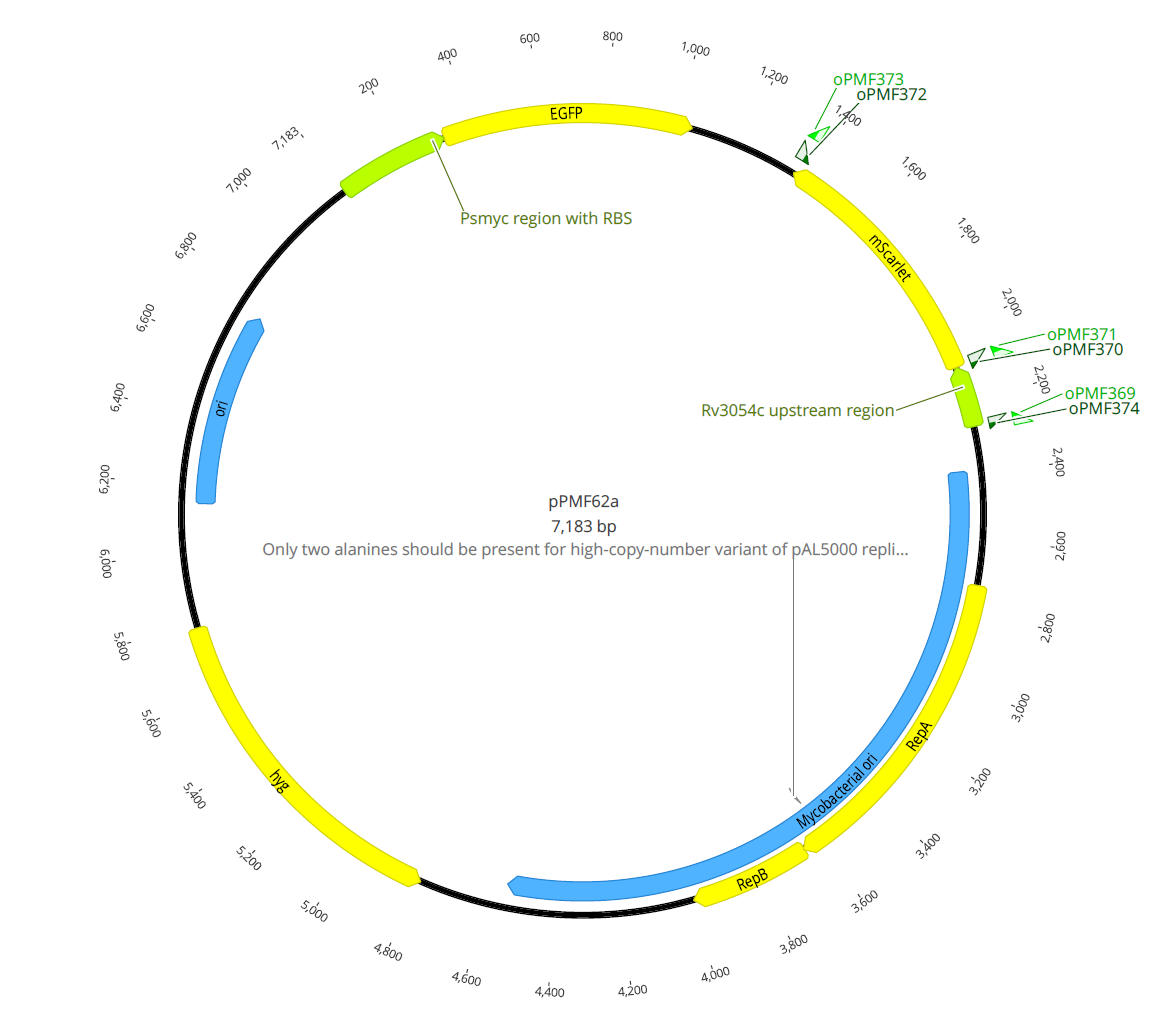


**S1 Figure:** **Vector map for the reporter plasmid pPMF62a**.

Supplement: S1 Fig — (DOCX) [file pbio.3002852.s012.docx]
