# Supplementary material for: Environmental fungi target thiol homeostasis to compete with Mycobacterium tuberculosis
Source: PLoS Biol. 2024 Dec 3;22(12):e3002852. doi: 10.1371/journal.pbio.3002852 (PMC11614215; doi:10.1371/journal.pbio.3002852)
Supplement: S2 Fig — (DOCX) [file pbio.3002852.s013.docx]

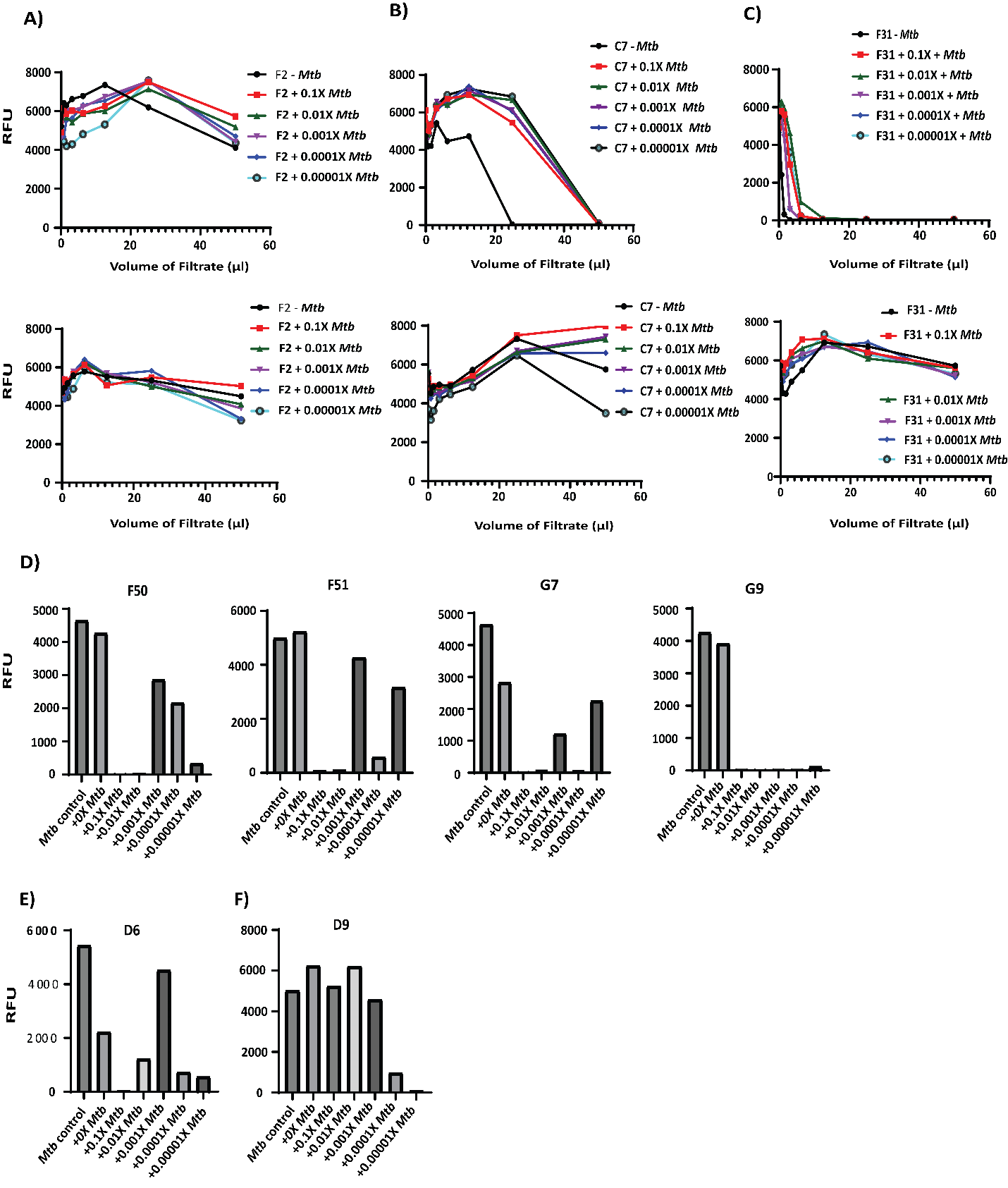




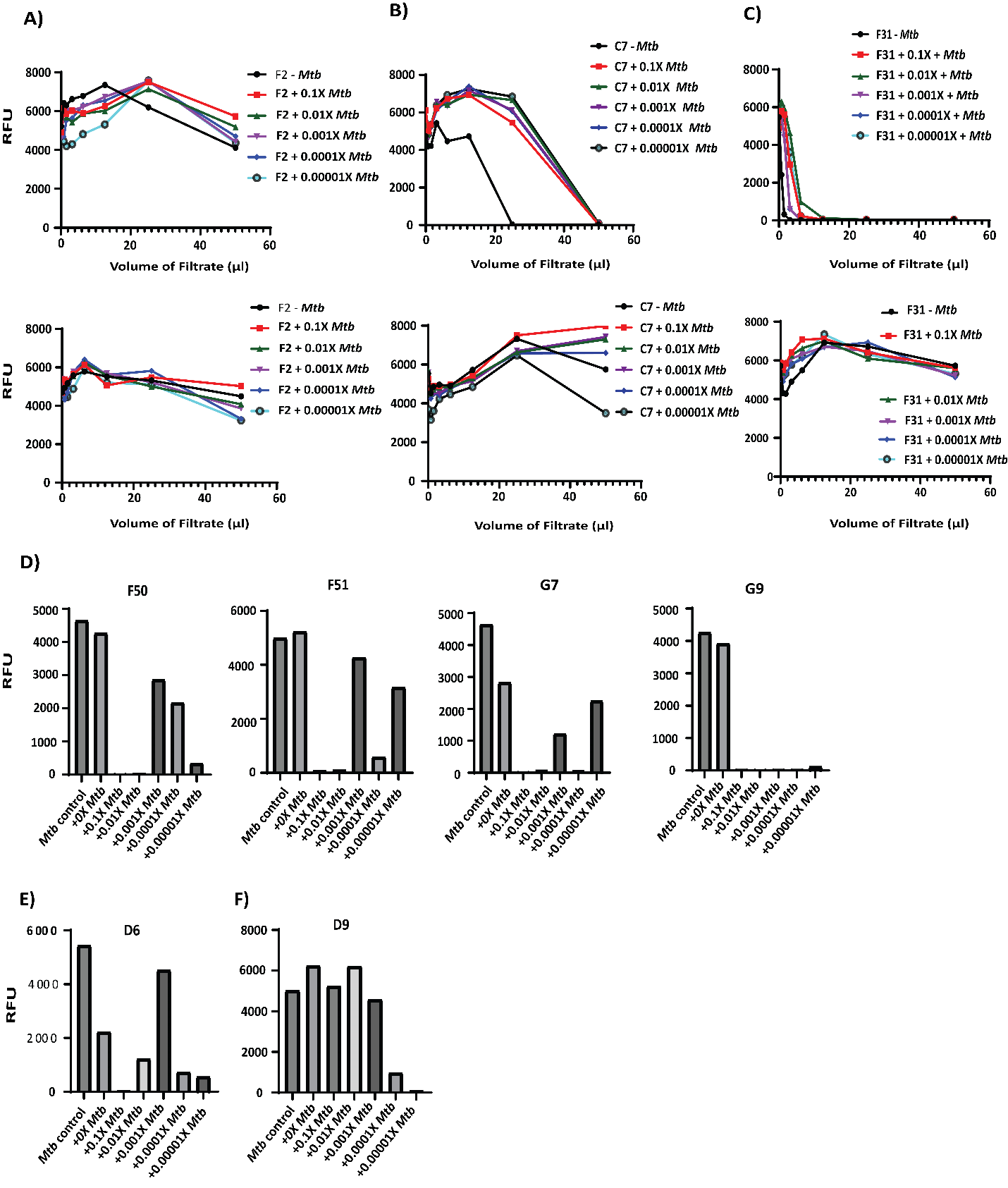


**S2 Fig.: Growth inhibition activity assays in different media.** Growth Inhibition of mScarlet *Mtb* H37Rv using filtrates from mono-cultures and co-cultures with different concentration of inducer *Mtb* H37Rv in Czapek Dox Media (Top) and Yeast Extract Media, YEM (Bottom) for **A)** F2, **B)** C7 and **C)** F31 fungus. **D)** Growth inhibition of mScarlet *Mtb* H37Rv using filtrates from mono- and co-culture of F2-like fungi (F50 and F51). Growth Inhibition using other potential hits **E)** D6 and **F)** D9 showing induced activity against mScarlet H37Rv. mScarlet RFU at the best MIV with respect to one inducer *Mtb* concentration shown for **D)**, **E)** and **F)**. Underlying data can be found in the supplemental file “S1_Data”.
