# Supplementary material for: Environmental fungi target thiol homeostasis to compete with Mycobacterium tuberculosis
Source: PLoS Biol. 2024 Dec 3;22(12):e3002852. doi: 10.1371/journal.pbio.3002852 (PMC11614215; doi:10.1371/journal.pbio.3002852)
Supplement: S3 Fig — (DOCX) [file pbio.3002852.s014.docx]

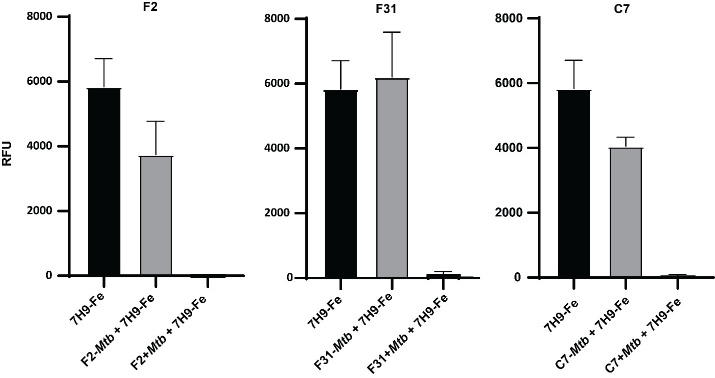


**S3 Fig.: No siderophore activity in fungal filtrates.** Mono- and co-culture derived fungal filtrates were used to perform growth inhibition assay for mScarlet *Mtb* H37Rv in an iron-free 7H9+ADGNTw media supplemented with 50 µg/mL hygromycin and 250 µM ferric ammonium citrate. Siderophore activity in terms of RFU of mScarlet *Mtb* H37Rv for F2-*Mtb* and F2 + 0.1X *Mtb* filtrates (left panel), C7-*Mtb* and C7 + 0.01X *Mtb* filtrates (middle panel), F31-*Mtb* and F31 + 0.0001X *Mtb* filtrates (right panel) in iron supplemented media. mScarlet RFU at the best MIV with respect to one inducer *Mtb* concentration are presented above (n=2). Underlying data can be found in the supplemental file “S1_Data”.
