# Supplementary material for: Environmental fungi target thiol homeostasis to compete with Mycobacterium tuberculosis
Source: PLoS Biol. 2024 Dec 3;22(12):e3002852. doi: 10.1371/journal.pbio.3002852 (PMC11614215; doi:10.1371/journal.pbio.3002852)
Supplement: S4 Fig — (DOCX) [file pbio.3002852.s015.docx]

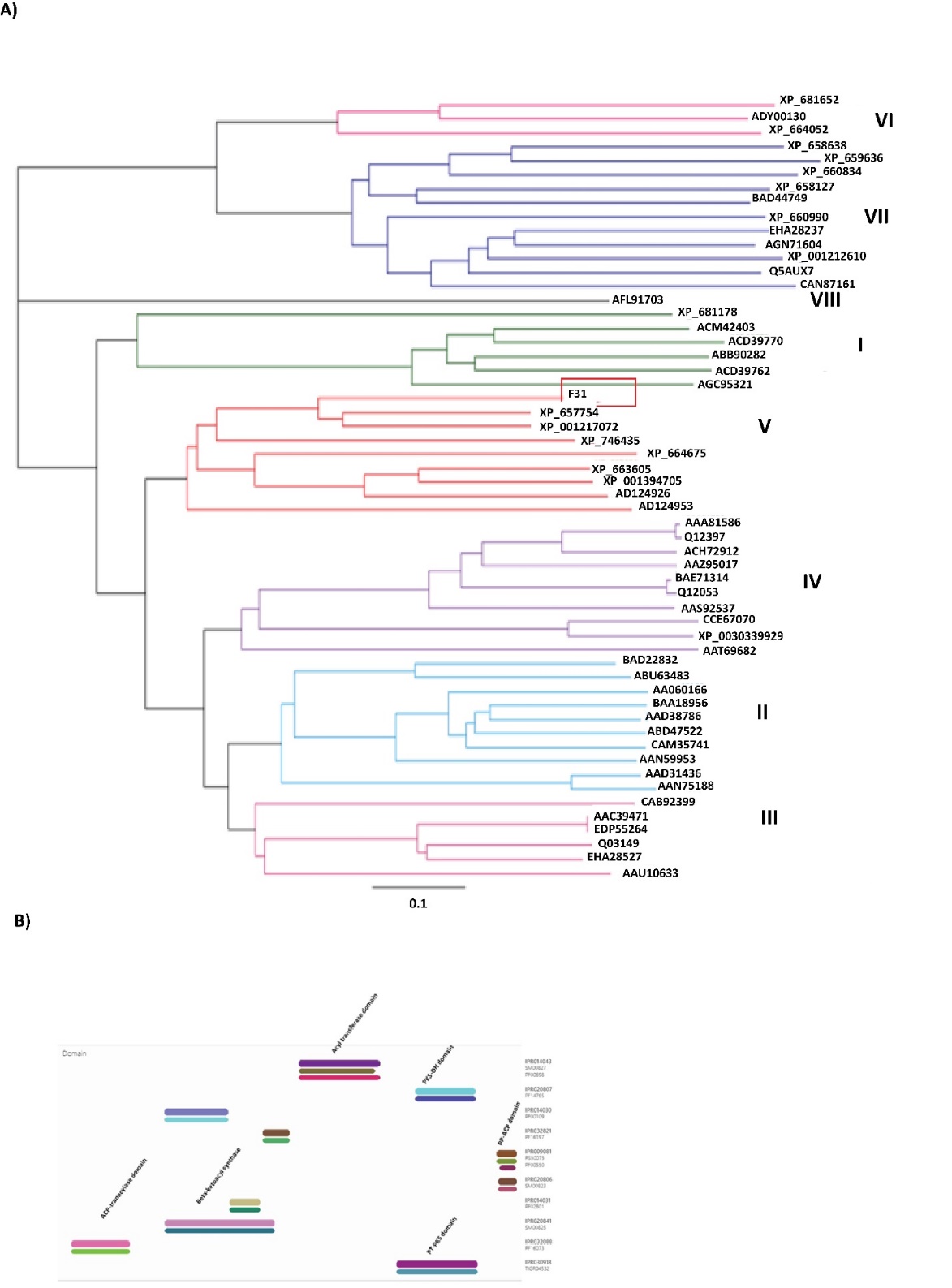


**S4 Fig.: Phylogenetic and protein characterization of F31_005372.** **A)** Phylogenetic analyses for F31_005372 placement (enclosed in red rectangle) amongst 55 NR-PKS using Neighbor-Joining (NJ) method in Geneious Prime **B)** Domain architecture of F31_005372. InterPro Scan analysis for domain prediction was performed for the Type I NR-PKS (F31_005372) using the protein sequence. ACP: Acyl Carrier Protein domain; PKS: polyketide synthase; PP: phosphopatathiene domain; PT: product template domain; DH: dehydrogenase domain. Underlying data can be found in the supplemental file “S1_Data”.
