# Supplementary material for: Environmental fungi target thiol homeostasis to compete with Mycobacterium tuberculosis
Source: PLoS Biol. 2024 Dec 3;22(12):e3002852. doi: 10.1371/journal.pbio.3002852 (PMC11614215; doi:10.1371/journal.pbio.3002852)
Supplement: S5 Fig — (DOCX) [file pbio.3002852.s016.docx]

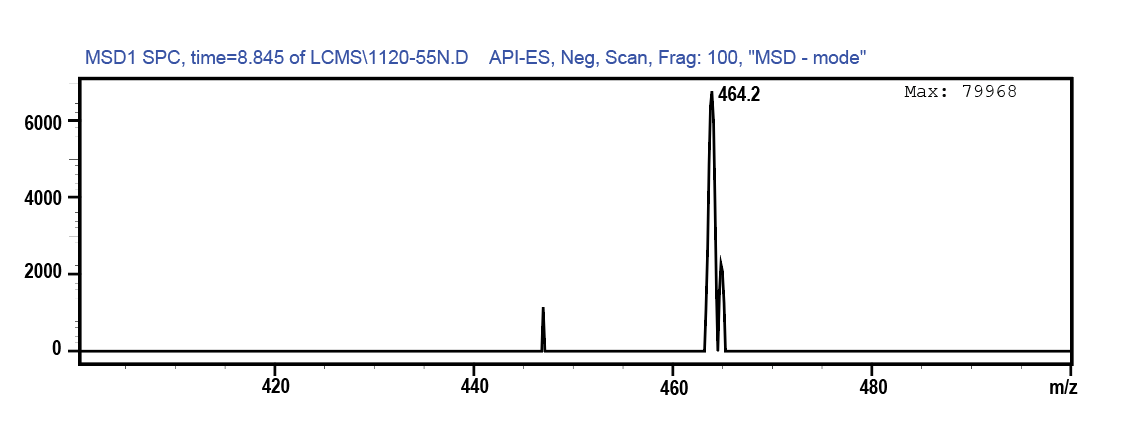

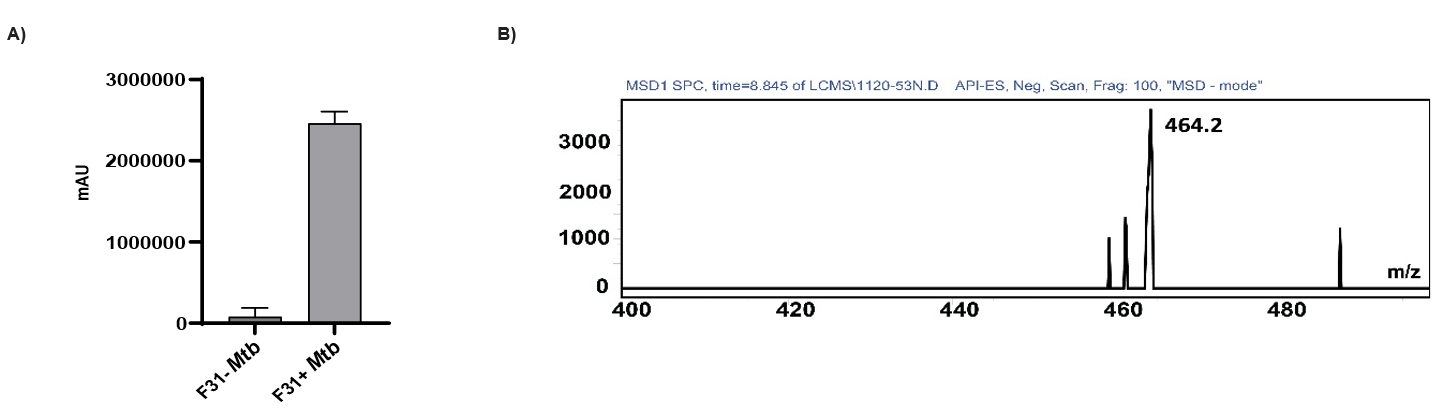


**C)**
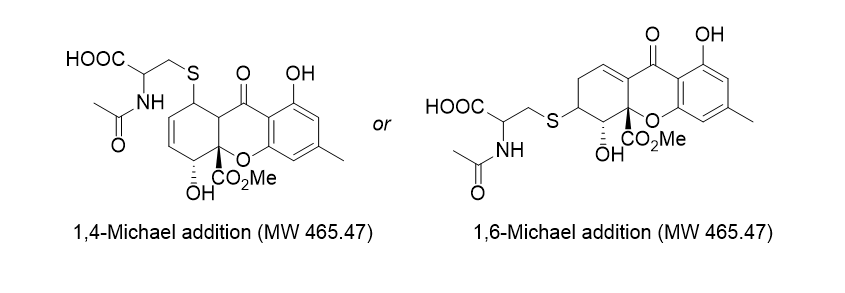


**S5 Fig.:**  N**idulalin A detection in cell-free supernatant** **A)** Detection of 303 (m/z) ion representing nidulalin A in F31-*Mtb* /F31+*Mtb* filtrates by LC-MS/MS positive ion mode. **B)** Nidulalin A-mycothiol adduct formed in *Mtb* H37Rv upon treatment with 10X MIC concentration of nidulalin A for 24 h detected by LC-MS as the mercapturic acid covalent conjugate (m/z 464.2 Da) in the extracellular milieu. **C)** Probable structures of the nidulalin A adduct. Underlying data can be found in the supplemental file “S1_Data”.
