# Supplementary material for: Environmental fungi target thiol homeostasis to compete with Mycobacterium tuberculosis
Source: PLoS Biol. 2024 Dec 3;22(12):e3002852. doi: 10.1371/journal.pbio.3002852 (PMC11614215; doi:10.1371/journal.pbio.3002852)
Supplement: S6 Fig — (DOCX) [file pbio.3002852.s017.docx]

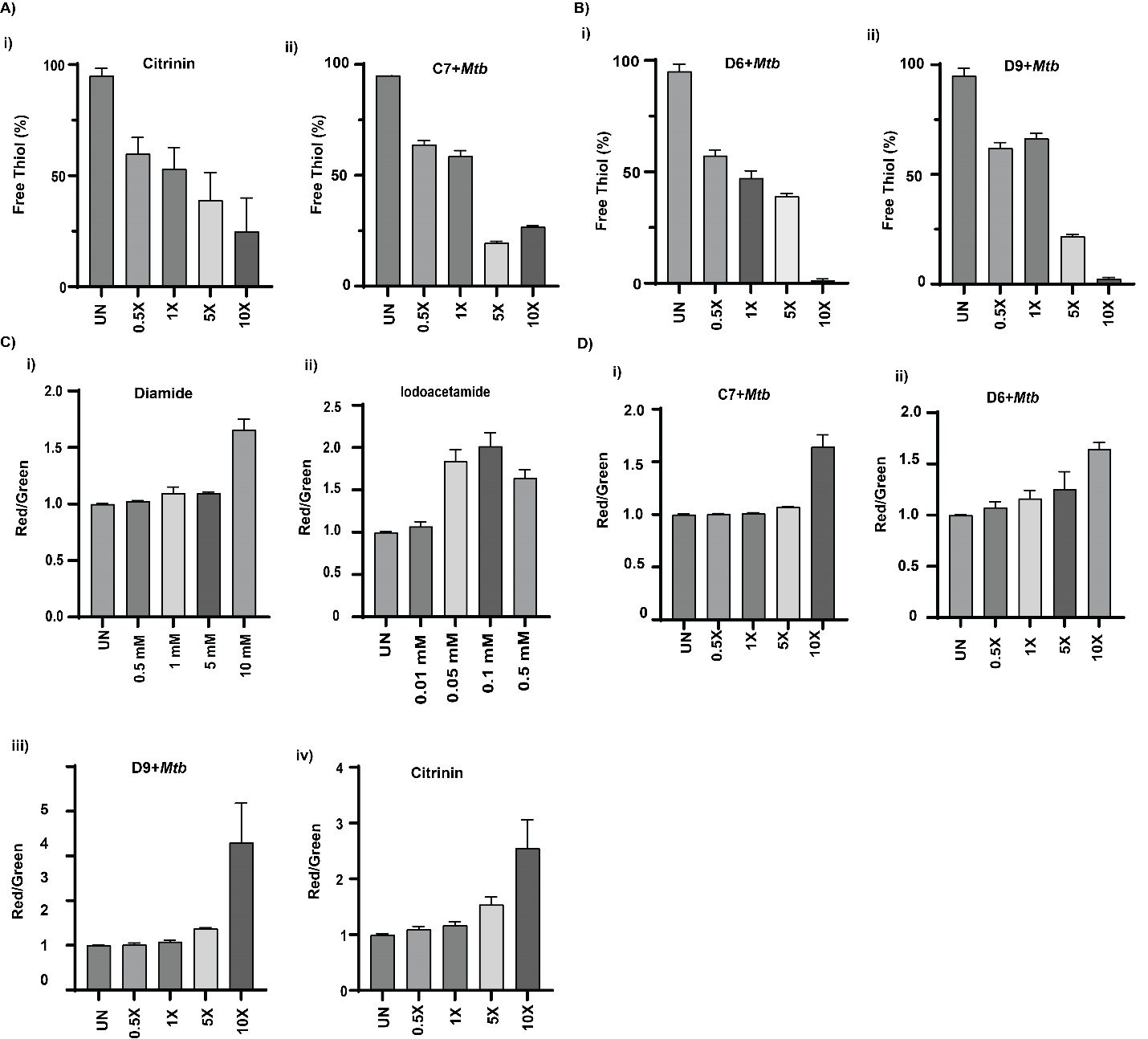


**S6 Fig.: Thiol-reactive oxidative stress in *Mtb* H37Rv in response to induced fungal products. A)** Free thiol depletion in *Mtb* H37Rv cells exposed to different concentrations of (i) citrinin and (ii) C7+*Mtb* filtrate; **B)** Free thiol depletion in *Mtb* H37Rv cells exposed to other filtrates like (i) D6+*Mtb* or (ii) D9+*Mtb* filtrates. **C)** Red/Green ratio representing *Rv3054c* expression upon treatment with (i) diamide (0-10 mM) and (ii) iodoacetamide (0-0.5 mM). **D)** Red/Green ratio upon treatment with (i) C7+*Mtb* (ii) D6+*Mtb* (iii) D9+*Mtb* filtrates and (iv) purified citrinin at 0-10X MIV/MIC concentrations. Underlying data can be found in the supplemental file “S1_Data”.
