# Supplementary material for: Environmental fungi target thiol homeostasis to compete with Mycobacterium tuberculosis
Source: PLoS Biol. 2024 Dec 3;22(12):e3002852. doi: 10.1371/journal.pbio.3002852 (PMC11614215; doi:10.1371/journal.pbio.3002852)
Supplement: S7 Fig — (DOCX) [file pbio.3002852.s018.docx]

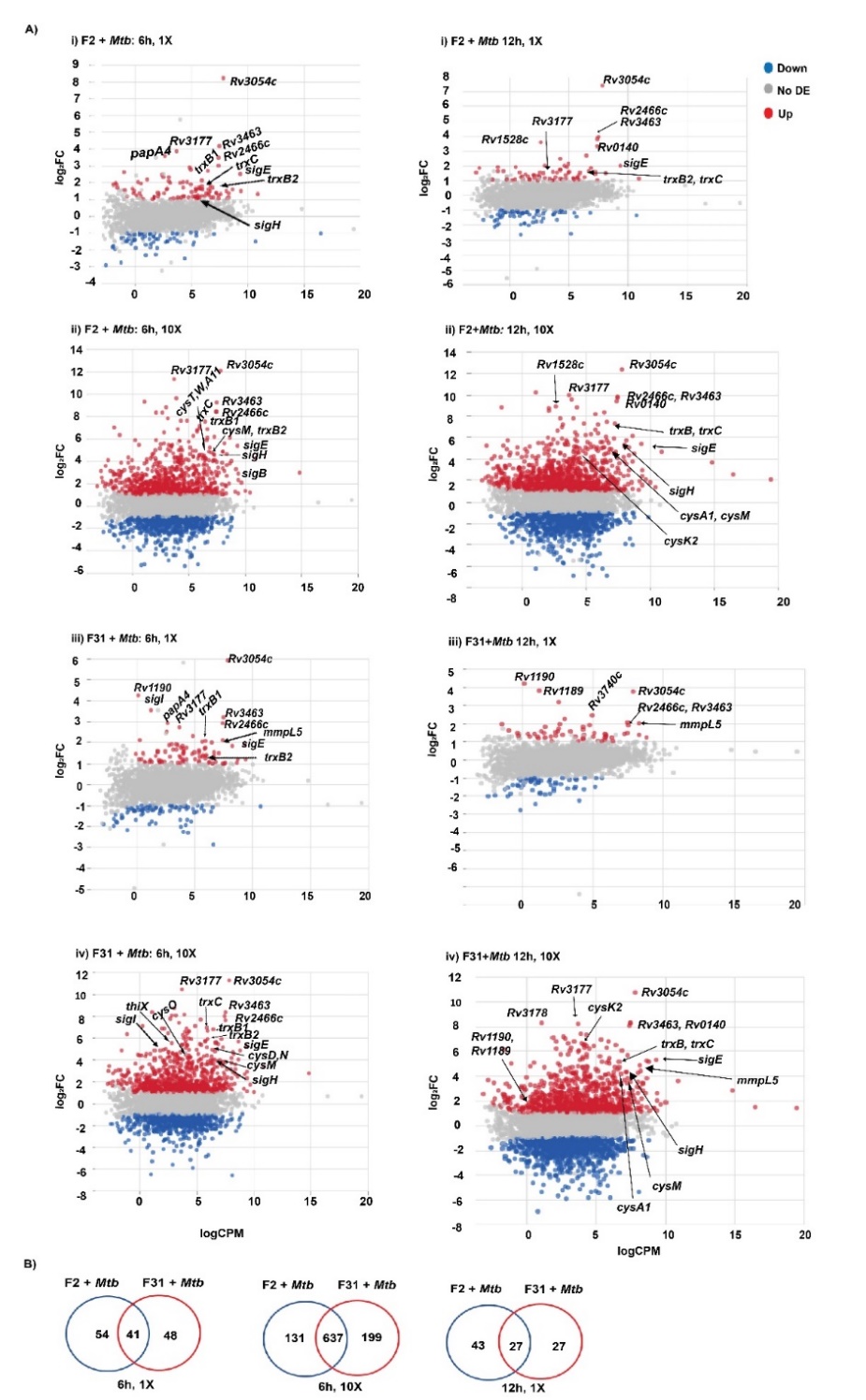


**S7 Fig.:** **Thiol stress response in *Mtb***. **A)** MA plots showing overexpression of *Mtb* genes involved in thiol stress upon exposure to F2+*Mtb* and F31+*Mtb* filtrates after 6h (left 1-iv) and 12h (right i-iv) treatment at 1X and 10X MIVs. Red dots represent the upregulated *Mtb* genes; blue dots are downregulated genes, and the gray dots are non-differentially expressed genes. **B)** Venn diagram representing the overlap of upregulated genes between F2+*Mtb* and F31+*Mtb* exposed cells at different treatment conditions. Underlying data can be found in the supplemental files “S1_Data” and “S3_Data”.
