# Supplementary material for: Environmental fungi target thiol homeostasis to compete with Mycobacterium tuberculosis
Source: PLoS Biol. 2024 Dec 3;22(12):e3002852. doi: 10.1371/journal.pbio.3002852 (PMC11614215; doi:10.1371/journal.pbio.3002852)
Supplement: S8 Fig — (DOCX) [file pbio.3002852.s019.docx]

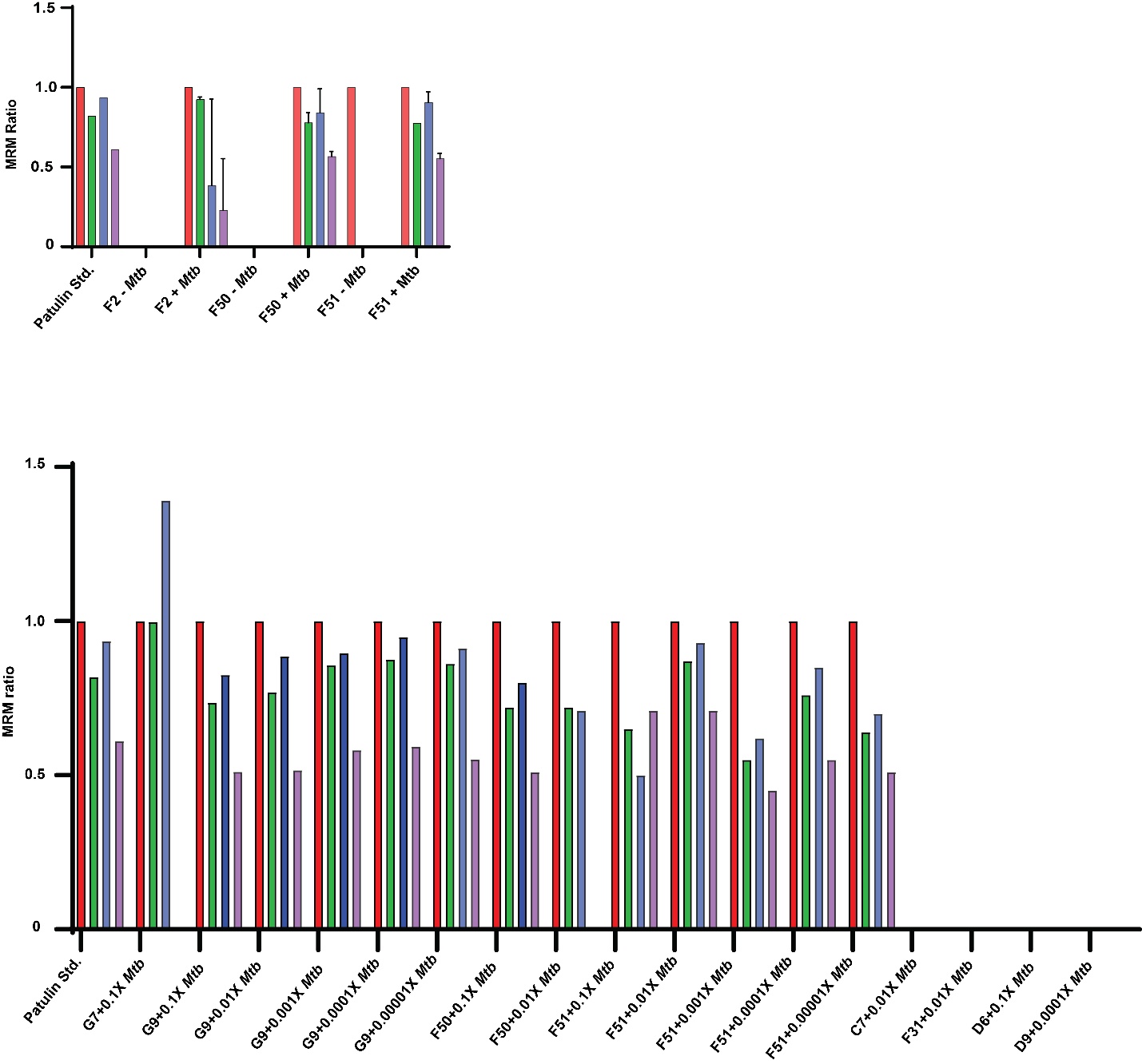


**S8 Fig.: Patulin scanning in induced fungal filtrates.** MRM scan to indicate the patulin presence in F2+*Mtb*, F50+*Mtb* and F51+*Mtb* co-culture samples as detected by LC-MS/MS by quantifying the fragment abundance for four product ions (109, 99, 81, 71) obtained when patulin standard, M+H^+^ precursor (m/z 155) was hit with 9,7,10, and 10V collision energies respectively. Underlying data can be found in the supplemental file “S1_Data”.
