# Supplementary material for: Environmental fungi target thiol homeostasis to compete with Mycobacterium tuberculosis
Source: PLoS Biol. 2024 Dec 3;22(12):e3002852. doi: 10.1371/journal.pbio.3002852 (PMC11614215; doi:10.1371/journal.pbio.3002852)
Supplement: S9 Fig — (DOCX) [file pbio.3002852.s020.docx]

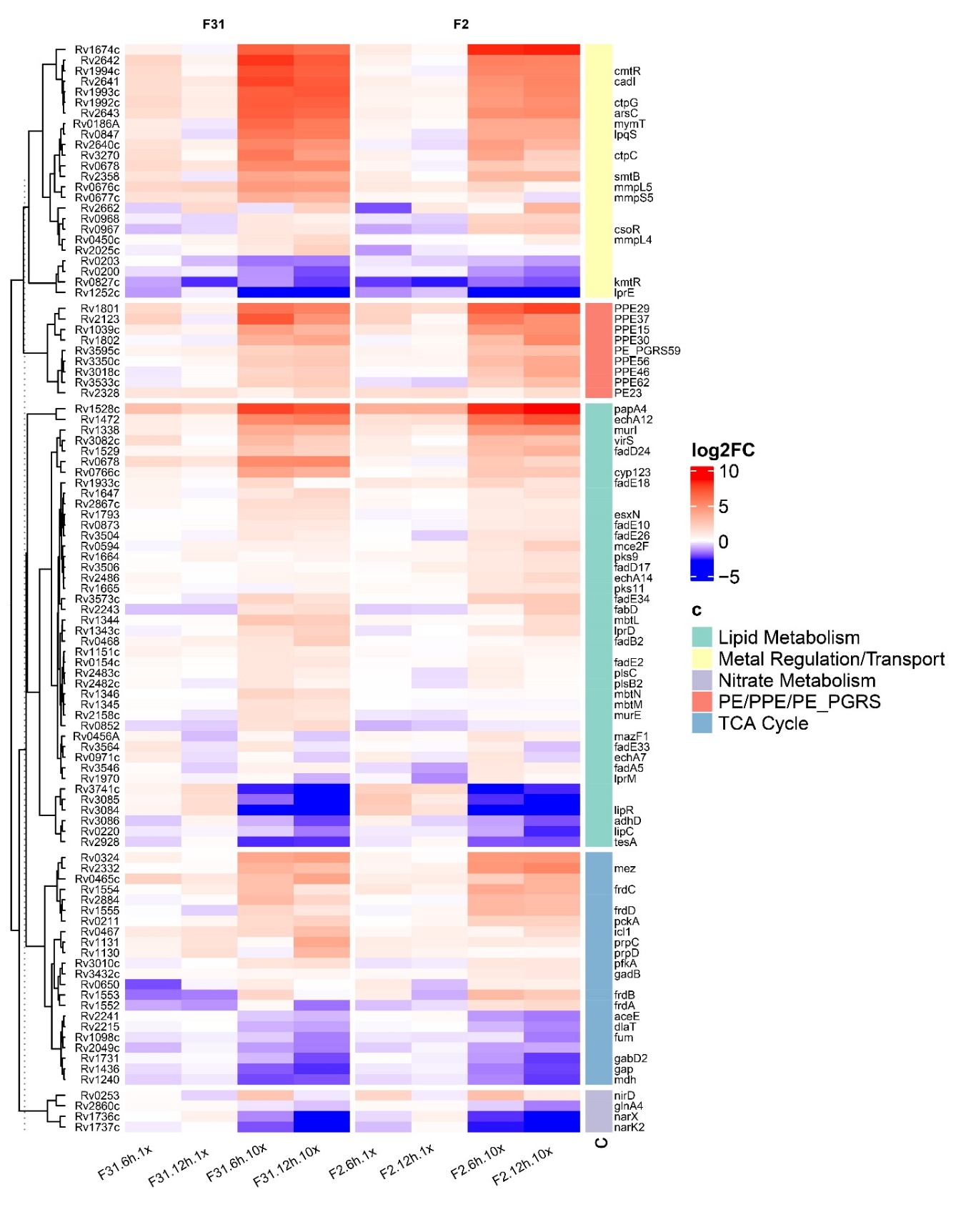


**S9 Fig.:** Heat map showing differential expression of genes involved in variety of pathways within *Mtb* upon treatment with F31+*Mtb* and F2+*Mtb* filtrate. Underlying data can be found in the supplemental files “S1_Data” and “S3_Data”.
