# Supplementary material for: Environmental fungi target thiol homeostasis to compete with Mycobacterium tuberculosis
Source: PLoS Biol. 2024 Dec 3;22(12):e3002852. doi: 10.1371/journal.pbio.3002852 (PMC11614215; doi:10.1371/journal.pbio.3002852)
Supplement: Attachment — Submitted filename: RTR_PlosBioBogs.docx [file pbio.3002852.s021.docx]

Response to reviewers, PLOS Biology Submission (PBIOLOGY-D-24-02662R1), “Environmental fungi target thiol homeostasis to compete with Mycobacterium tuberculosis".

Reviewer 1. We thank the reviewer for the positive comments.

Reviewer 2. Again, we thank reviewer for the positive comments and suggestions.

- fungus (singular) vs fungi (plural) - please ensure these are used correctly throughout the manuscript.

**Corrected throughout.**

- it is quite remarkable that only a single biosynthetic locus was induced with fungus F2. Just out of interest, what are some of the non-biosynthetic genes that are upregulated and is there any theme among the three strains here?

**It surprised us as well. Overall, though only about 100 or so genes were differentially expressed significantly in each sample in the co-culture compared to the monoculture. Among these three there were a couple of common genes, such as the glucose oxidase involved in increasing glycolysis and presumably the pool of activated CoA esters available for secondary metabolite production. All three strains also upregulated several annotated transporter systems.**

- It would be nice to add the structure of the mycothiol-trapped nidulanin (Fig. S5). Even if the authors do not have conclusive NMR evidence, a proposed structure could be added. The authors may also consider moving parts or all of Fig. S5 to the main text, as this is a key figure. Just a suggestion, ultimately at the discretion of the authors.

**We have added the adduct structures but we did not conclusively determine if the 1,4 or 1,6 conjugate addition was actually produced so we feel like this should still remain in the supplemental.**

- "In this microenvironment, which appears to support the highest concentration of mycobacteria found anywhere in nature..." I think "known" or "thus far" needs to be added somewhere in this sentence, as clearly all natural environments/microenvironments have not been investigated.

**As suggested.**

- do the authors have any clues regarding the nature of the elicitor (from Mtb) for these three fungal products?

**This is an interesting question. At this point we know that heat-killed organisms don’t induce expression, but we’ve just started to tease apart what the inducer actually is. Its most likely one of the TB-specific glycolipids but sorting out which is on-going.**
